# Oral health‐related behaviours do not mediate the effect of maternal education on adolescents' gingival bleeding: A birth cohort study

**DOI:** 10.1111/cdoe.12350

**Published:** 2017-11-27

**Authors:** Marco A. Peres, Gustavo G. Nascimento, Karen G. Peres, Flavio F. Demarco, Ana B. Menezes

**Affiliations:** ^1^ Australian Research Centre for Population Oral Health Adelaide Dental School The University of Adelaide Adelaide SA Australia; ^2^ Graduate Program in Dentistry Federal University of Pelotas Pelotas Brazil; ^3^ Section of Periodontology Department of Dentistry and Oral Health Aarhus University Aarhus Denmark; ^4^ Graduate Program in Epidemiology Federal University of Pelotas Pelotas Brazil

**Keywords:** adolescent, cohort studies, epidemiology, gingival bleeding, life course, marginal structural modelling, mediation analysis, oral behaviours, oral heath inequalities, path analysis

## Abstract

**Objectives:**

To test whether maternal education has a direct effect on gingival bleeding in adolescents aged 12 and to assess whether oral health behaviours over time mediate that association.

**Methods:**

Two oral health studies nested in the 1993 Pelotas (Brazil) birth cohort study were carried out in participants aged 6 (n = 359) and 12 years (n = 339). The proportion of teeth with bleeding on probing (BOP) and the median number of teeth with gingivitis at age 12 were recorded. Maternal education at birth was the exposure. Toothbrushing frequency and dental visit at ages 6 and 12 years were investigated as mediators of the association between maternal education at birth and gingival bleeding. Time‐varying family income through childhood and adolescence was included as later confounder. Paternal education was taken as baseline confounder. The controlled direct effect (CDE) of maternal education at child's birth on gingival bleeding at age 12 was estimated using marginal structural models (MSM). Additionally, path analysis was employed to estimate standardized direct, indirect and total effects of maternal education at birth on gingival bleeding.

**Results:**

Adjusted analyses using MSM showed that adolescents whose mothers had <8 years of education had 3.82 higher risk of having teeth with gingival bleeding above the median (rate ratio RR 3.82; 95% CI: 1.68‐8.19). Low maternal education doubled the proportion of gingival bleeding at age 12 not mediated by dental visit and toothbrushing frequency (RR 1.99; 95% CI: 1.52‐2.60). Path analysis revealed that maternal education had a direct effect on gingival bleeding independently of the mediators.

**Conclusions:**

The pattern of oral health behaviours does not explain the association between mother's education and adolescent's gingival bleeding. Individual‐based approaches focused on oral health‐related behaviours tend to fail to prevent gingival bleeding.

## 1 INTRODUCTION

Gingival bleeding may be considered a marker of chronic gingival inflammation^1^; it is common among children and adolescents,^2^ and it is associated with periodontitis.^3^ Several epidemiological studies have reported an association between adolescents' gingival bleeding and adverse socioeconomic conditions,^4^, ^5^ inadequate toothbrushing and flossing^6^ and lack of dental attendance.^7^

Most theoretical models regarding the effect of socioeconomic position on periodontal health state that the main connection between the two is via oral health‐related behaviours.^8^, ^9^ However, there are some important theoretical, methodological and statistical limitations in the most commonly adopted approaches. Socioeconomic position is frequently assessed cross‐sectionally or relied upon retrospective reports about past socioeconomic circumstances. The ideal design to address the impact of different life course socioeconomic circumstances on health, including the role of potential mediators, is a birth cohort study.

Among several measures of socioeconomic position used in epidemiological studies, maternal education is one of the most popular, particularly when children and adolescent's health outcomes are of interest. Even in high‐income countries with high levels of human development and gender equity, such as Scandinavian countries, mother‐related behavioural factors tend to be more closely associated with their educational attainment, as children's health behaviour. On the other hand, fathers are more likely to work full time and have higher earnings than mothers.^10^ It is postulated that educated mothers are more involved in their children's activities, more closely monitor their children's behaviours, more likely to be health conscientious and have better health knowledge than less educated mothers.^11^ Findings from a large nationally representative birth cohort study from the United States revealed that educated mothers were most likely to practise more advantageous health investment behaviours throughout early childhood, including more preventive care, nutrition, safety issues, physical activity and less television watching.^12^ A birth cohort study conducted in Australia showed similar findings.^13^ An interesting study from China examined the effect of maternal education on the health of young children using a large sample of adopted children. The main effect of the level of mother's education on children's health was identified as through postnatal nurturing, suggesting that mothers' influence on children's health is extended beyond biology.^14^

From a life course perspective, theories have been formulated to explain how various biological, behavioural and social factors experienced during the life course influence health and disease later on. Essentially, there are 2 main proposals but with different subclassifications. The first is the critical period model (also known as programming), which states that there is a specific period of time—perinatal and childhood—when a given exposure is crucial to cause permanent and irreversible health damage. The second is the accumulation of risk model, which occurs when negative exposures early in life combine with adverse exposures later in life producing an additive effect on health in the future.^15^ The principle here is that risk accumulates gradually. Regardless of the proposed conceptual framework, there is no dispute that psychosocial factors are mediators of the association between the social conditions (distal determinants) and health (the outcome). For example, toothbrushing frequency is determined by socioeconomic conditions and may influence dental outcomes such as dental caries and periodontal health.^16^, ^17^ Epidemiologically speaking, a mediator is defined as a variable that occurs in a causal pathway from a distal cause to an outcome. It causes variation in the outcome and itself is determined by the original causal variable.^18^ Therefore, in order to longitudinally investigate the complex and interconnected relationship between different levels of health determinants is mandatory to assess mediation by combining the use of solid theoretical basis and appropriate statistical tools.

Conventional regression methods have been criticized as insufficient tools to deal with mediation given that these methods conditioning on mediator may create collider bias^19^ even after adjustment for past confounders.^20^ In addition, they cannot assess time‐varying mediators and confounders. Alternatives techniques, which have been proposed to overcome these limitations, include marginal structural models (MSM) and path analysis. Briefly, marginal structural models (MSM) are a class of causal models for the estimation of causal inference using observational data of a time‐varying exposure (such as family income over the life course) in the presence of time‐varying variables that may be simultaneously confounders and mediators (such as oral health behaviours in the life span).^20^ Path analysis is a statistical method that allows the assessment of complex causal relationships between one or more independent variables and one or more dependent variables.^21^

Accordingly, this study aimed to answer the following research questions: (i) Is there an effect of life course socioeconomic circumstances on adolescents' gingival bleeding? (ii) Do oral health‐related behaviours mediate the relationship between life course socioeconomic circumstances and adolescents' gingival bleeding? (iii) Is there a direct effect of early life socioeconomic circumstances on adolescents' gingival bleeding? and (iv) Is there consistency between estimates from MSM and path analysis?

## 2 METHODS

### 2.1 The 1993 Pelotas birth cohort

**Figure 1:**
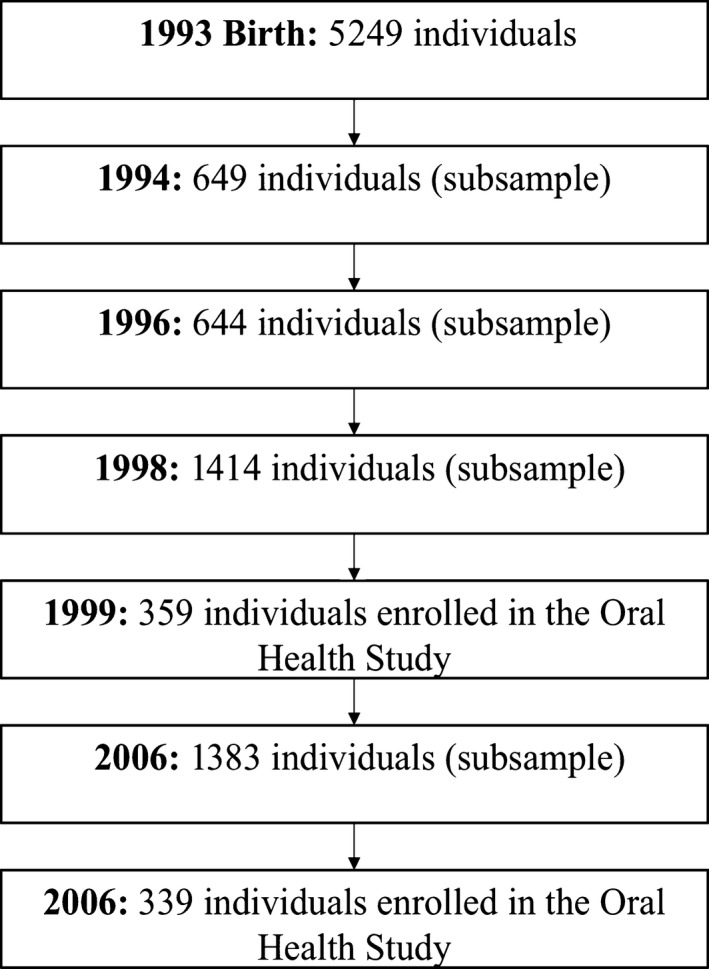
Flow chart of the 1993 Pelotas birth cohort

This study is nested in the 1993 Pelotas (Brazil) population‐based birth cohort study. Detailed methodology aspects of the cohort have been published elsewhere.^22^ Briefly, all hospitals in Pelotas were visited daily from 1 January to 31 December 1993. All mothers who had given birth at these institutions were invited to take part in the study. The children included in the cohort (n = 5249) represent 99% of the total births in Pelotas. All mothers were asked questions on their social and economic conditions, demographics, pregnancy characteristics, health‐related behaviours, health care and morbidity, and the children were weighed, measured and examined at birth. Figure 1 depicts the flow chart of the 1993 Pelotas birth cohort.

A subsample of the cohort children was selected and visited in the subsequent follow‐up waves, at ages 1 (n = 649), 3 (n = 644), 6 (n = 1414) and 12 (n = 1383) months. In 1998, a sample of the original cohort, including all low birthweight children and a random 20% of the remainder, was revisited. Of 1460 eligible children, 87% (1270 children) were located.

### 2.2 Oral health study at age 6

A subsample, drawn from the 1998 followed‐up group, was examined to estimate the occurrence of several oral health outcomes,^23^, ^24^ resulting in a sample of 400 children. As low birthweight children were overrepresented in the oral study sample (29.7% compared with 10% in the original cohort), all analyses were weighted. The weights used were 0.34 (0.10/0.297) for low birthweight children.

The oral health study included a dental examination (caries, malocclusion, oral mucosa lesions) and a questionnaire. This was administered to the participants' mothers at home and included, among others, questions related to dental hygiene habits, such as age when the child started brushing teeth, current toothbrushing daily frequency, use of toothpaste and dental floss and type and use of dental services. Pretest of the questionnaire, examiner calibration of 3 dentists and a pilot study were carried out before the field work. The participation rate was 89.7% (n = 359).

### 2.3 Oral health study at age 12

All of the 359 children who participated in the study when they were 6 years old were visited in their homes in 2005, when they were 12 years old (Figure 1). A questionnaire was applied to the adolescents, containing questions on the use of dental services, history of toothache, and habits and behaviours related to oral hygiene. Four teams of fieldworkers were formed, each consisting of an examiner and an interviewer. Headlights were used to improve the field of view. Gingival bleeding was assessed 10 seconds after probing at 6 sites in all teeth (mesio‐buccal, mid‐buccal, disto‐buccal, disto‐lingual, mid‐lingual and mesio‐lingual) using a ball‐ended periodontal probe.^25^

### 2.4 Outcome—gingival bleeding at age 12

Gingival bleeding was used in 2 ways: (i) the proportion of teeth with gingival bleeding, calculated by dividing the number of teeth with bleeding by the number of examined teeth; (ii) the dichotomous variable based on the median number of teeth with gingival bleeding (5 teeth; <5 teeth with gingival bleeding [reference category] vs more than 5 teeth with gingival bleeding).

### 2.5 Main exposure—maternal education at the time of the child's birth

Maternal education at child's birth was assessed considering full years of formal schooling at the child's birth. This information was converted into a dichotomous variable as follows: 9 or more years of education (reference category) vs 8 or less years of education.

### 2.6 Covariates

Paternal education at child's birth was considered as a baseline confounder of the relationship between maternal education and gingival bleeding. Paternal education considered full years of formal schooling at the child's birth (9 or more years of education vs 8 or less years of education). Family income information at birth and at ages 4 and 11 were gathered in Brazilian minimum wages (MW) at the time of data collection. The variable was then dichotomized into more than 2.5 MW (reference category) vs 2.5 MW or less. Each measure of family income was used as later confounder in the relationship between maternal education at child's birth and gingival bleeding at age 12. The age when the child started toothbrushing was included in the model as an exposure‐mediator variable. The variable was dichotomized into started toothbrushing when the first tooth was erupted (reference category) vs started toothbrushing after the first tooth was erupted.

### 2.7 Mediators

Based on the literature, 2 time‐varying mediators were considered in the proposed model: (i) frequency of toothbrushing at ages 6 and 12 (2 or more times a day vs <2 times a day and (ii) dental visit in the last year at ages 6 and 12 (yes vs no).

### 2.8 Conceptual framework and analytical approach

**Figure 2:**
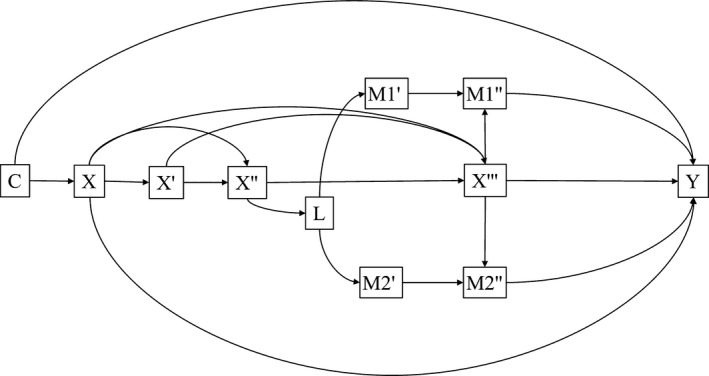
Directed acyclic graph of the relationship between maternal schooling at child's birth and gingival bleeding at age 12. C = Paternal schooling at child's birth; X = maternal schooling at child's birth; X′ = family income at birth; X″ = family income at age 4; X‴ = family income at age 11; L = age when the child started toothbrushing; M1′ = toothbrushing at age 6; M1″ = toothbrushing at age 12; M2′ = dental visit at age 6; M2″ = dental visit at age 12; Y = gingival bleeding at age 12. Arrows between C and all other variables were omitted for ease of interpretation. 1993 Pelotas birth cohort study; Pelotas; Brazil

We described the studied sample characteristics, according to the proportion of bleeding teeth, and maternal schooling at child's birth. Figure 2 depicts the proposed relationship between maternal schooling at child's birth and gingival bleeding at age 12.

In this study, different analytical approaches were used to assess mediation. Firstly, we estimated the controlled direct effect (CDE) of maternal education at child's birth on gingival bleeding at age 12 using marginal structural models (MSM). The CDE is a comparison of the expected outcome conditional on the exposure and the mediator for different values of the exposure while keeping the mediator fixed.^26^ To estimate the controlled direct effect of maternal schooling at child's birth on gingival bleeding not mediated by each dental behaviour (where M = 0 was set in the models), we used separate marginal structural models for each mediator, despite the knowledge that there would be combined effects. The final marginal structural model was controlled for both mediators. We tested both multiplicative and additive interactions (relative excess risk due to interaction) between exposure and each mediator. Details about interactions are presented in the Table S1. In the MSM approach, we estimated the stabilized inverse probability weight (SW) for the exposure and each mediator accordingly.^20^ Predicted probabilities for the numerator and denominator were assigned and later divided to obtain the stabilized weights.^20^ Final stabilized weights were obtained by multiplying the weight of the exposure by the weight of each mediator at a time. Further details regarding the steps used to estimate the SW are described in the Appendix S1.

Following the same relations depicted in Figure 2, path analysis was employed. The maximum‐likelihood estimation method was used in all models. Standardized direct, indirect and total effects on gingival bleeding were estimated. The Comparative Fit Index (CFI), the Tucker Lewis Index (TLI) and the root‐mean‐square error of approximation (RMSEA) were used to evaluate the fit of analytical models: 0.95 was taken as a minimum value for inferring model fit, according to the CFI and TLI indices,^27^ while RMSEA values from zero to 0.08 were accepted as indicative of fit.^28^ Modification indices were estimated to examine alternative models providing potentially better indices of fit. Whenever better‐fitting models supported by theory and previous empirical findings were observed, these were implemented. Statistical significance was taken to be <5.0%. All analyses were conducted in the software Stata 14.0 (StataCorp., College Station, TX, USA).

### 2.9 Sensitivity analysis

In mediation analysis, it is recommended to take into account mediator measurement error and unmeasured confounding biases. When estimating the controlled direct effect, mediator measurement error is not as threaten as unmeasured confounding as the mediator is controlled.^29^ For this reason, sensitivity analysis was performed to test the assumption of no unmeasured confounder (*U*). Sensitivity parameters of *U* were specified according to the previous literature. We used the model proposed by VanderWeele^30^ to calculate the bias introduced by *U* that could invalidate the controlled direct effect of maternal schooling at child's birth on gingival bleeding at age 12. The formula and the parameters used are described in details in the Appendix S2.

### 2.10 Ethical issues

Consent for interviews and examinations was obtained and both projects (age 6 and 12 years) were approved by the Pelotas Federal University Ethics Committee. Adolescents who presented with dental treatment needs were referred to the Dental Clinic of the Graduate Program in Dentistry of Federal University of Pelotas.

## 3 RESULTS

Among 400 children selected to participate in the study, 359 (89.8%) were investigated at 6 years and 339 (84.7%) at 12 years of age (Figure 1). Families who moved out the city was the main reason for dropout. Overall, the median number of teeth with gingival bleeding was 5, and the mean percentage of teeth with gingival bleeding was 24. The mean and the proportion of participants with above median number of teeth with gingival bleeding were higher among those whose parents had had <8 years of education at birth (Table 1). Family income lower than 2.5 MW at birth and at ages 4 and 11 was also associated with more gingival bleeding. Toothbrushing less than twice a day and lack of dental visit at ages 6 and 12 were associated with more gingival bleeding (Table 1).

**Table 1:** Sample description (n, %, 95% CI) according to independent variables and outcomes

Variables	Sample n (%)	Mean gingival bleeding	95% CI	Crude rate ratio^a^	95% CI	Dichotomous gingival bleeding (more than 5 teeth)	95% CI	Crude risk ratio^b^	95% CI
Maternal schooling at child's birth									
9 or more years	78 (23)	14.5	11.1‐17.9	1.00	‐	24.7	14.8‐35.5	1.00	‐
8 or less years	261 (77)	27.3	24.6‐30.0	1.88	1.44‐2.45	54.4	48.3‐60.4	3.64	2.05‐6.45
Paternal schooling at child's birth									
9 or more years	87 (27.8)	26.7	23.8‐29.7	1.00	‐	30.2	20.3‐40.1	1.00	‐
8 or less years	226 (72.2)	30.2	21.2‐39.2	1.64	1.25‐2.13	53.1	46.5‐59.6	2.61	1.54‐4.43
Family income at child's birth									
2.5 or more MW	175 (51.6)	21.6	18.7‐24.6	1.00	‐	41.1	33.8‐48.5	1.00	‐
<2.5 MW	164 (48.4)	27.3	23.8‐30.8	1.26	1.00‐1.58	54.6	46.9‐62.3	1.72	1.11‐2.64
Family income at age 4									
2.5 or more MW	192 (57.8)	20.0	17.3‐22.6	1.00	‐	38.2	31.2‐45.2	1.00	‐
<2.5 MW	140 (42.2)	29.3	25.4‐33.1	1.46	1.16‐1.84	58.6	50.3‐66.8	2.28	1.46‐3.56
Family income at age 11									
2.5 or more MW	205 (60.4)	19.1	15.9‐22.4	1.00	‐	35.8	27.6‐44.0	1.00	‐
<2.5 MW	134 (39.5)	27.8	24.7‐30.8	1.45	1.15‐1.82	55.4	48.5‐62.3	2.22	1.42‐3.48
Age of toothbrushing start									
With the eruption of the first tooth	54 (17.0)	20.1	14.1‐26.2	1.00	‐	29.6	18.0‐43.6	1.00	‐
After the first tooth was erupted	264 (83.0)	24.3	21.8‐26.7	1.21	0.88‐1.64	48.7	42.4‐54.9	2.25	1.19‐4.23
Dental visit at age 6									
Yes	216 (64.3)	19.1	15.7‐22.4	1.00	‐	35.0	26.3‐43.6	1.00	‐
No	120 (35.7)	27.2	24.2‐30.2	1.43	1.13‐1.81	53.9	47.2‐60.2	2.17	1.37‐3.45
Dental visit at age 12									
Yes	243 (71.9)	21.2	18.7‐23.83	1.00	‐	40.3	34.1‐46.5	1.00	‐
No	95 (28.1)	32.4	27.9‐36.8	1.52	1.19‐1.95	66.3	56.6‐76.0	2.91	1.77‐4.78
Daily frequency of toothbrushing at age 6									
2 times or more	272 (82.9)	23.7	21.2‐26.2	1.00	‐	44.3	38.3‐50.2	1.00	‐
<2 times	56 (17.1)	26.2	20.6‐31.8	1.10	0.81‐1.50	55.3	41.9‐68.8	1.56	0.87‐2.78
Daily frequency of toothbrushing at age 12									
2 times or more	260 (79.3)	22.3	19.80‐24.8	1.00	‐	43.5	37.4‐49.5	1.00	‐
<2 times	68 (20.7)	30.9	25.4‐36.3	1.34	1.04‐1.84	60.3	48.4‐72.2	1.97	1.14‐3.40

^a^Rate ratio estimated by negative binomial regression for level the proportion of gingival bleeding as outcome.

^b^Risk ratio estimated by log‐linear regression for dichotomous gingival bleeding of gingivitis as outcome.

Table 2 presents the controlled direct effect of maternal schooling at child's birth on gingival bleeding estimated from MSM. When all mediators were appropriately controlled using MSM, adolescents whose mothers had <8 years of educational level had 3.82 times higher risk of having teeth with gingival bleeding above the median (RR 3.82; 95% CI: 1.70‐8.62) than those whose mothers had more than 9 years of education. Low maternal education was associated with a higher proportion of gingival bleeding at age 12 which was not mediated by dental visit and toothbrushing frequency (RR 1.99; 95% CI: 1.53‐2.60).

**Table 2:** Controlled direct effect from marginal structural models for prevalence and proportion of gingival bleeding. Pelotas, Brazil

	Dichotomous gingival bleeding (more than 5 teeth)	Proportion of gingival bleeding
Exposure	Risk ratio (95% CI)	Rate ratio (95% CI)
**Direct effect controlled for dental visit**		
Maternal schooling at child's birth		
9 or more years	1.00	1.00
8 or less years	3.71 (1.67‐8.26)	1.94 (1.49‐2.54)
**Direct effect controlled for daily frequency of toothbrushing**		
Maternal schooling at child's birth		
9 or more years	1.00	1.00
8 or less years	3.61 (1.70‐7.67)	2.07 (1.60‐2.70)
**Direct effect controlled for dental visit and for daily frequency of toothbrushing**		
Maternal schooling at child's birth		
9 or more years	1.00	1.00
8 or less years	3.82 (1.70‐8.62)	1.99 (1.53‐2.60)

CI, confidence interval.

**Figure 3:**
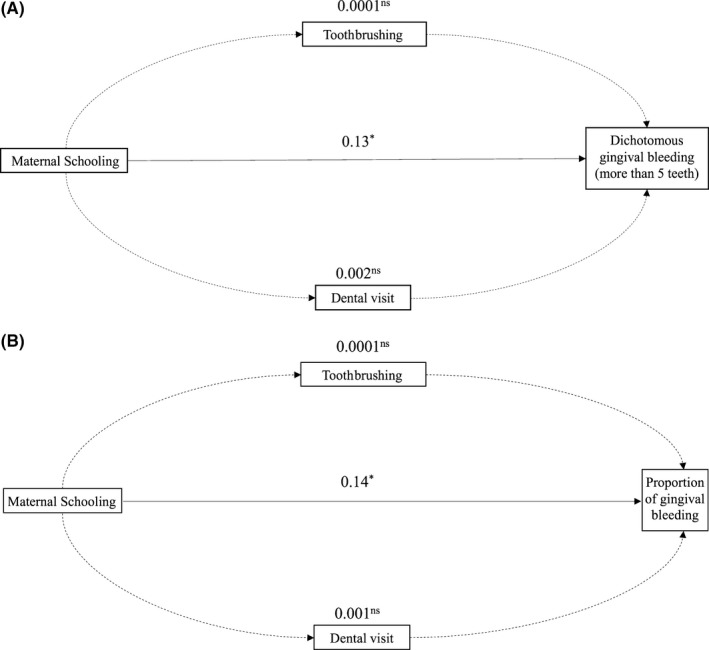
A, Path analysis of the effect of maternal schooling at child's birth on the dichotomous variable based on the median number of teeth with gingival bleeding (more than 5 teeth); (B) Path analysis of the effect of maternal schooling at child's birth on the proportion of teeth with gingival bleeding

Table 3 shows the effect of maternal education on gingival bleeding not mediated by frequency of toothbrushing and dental visit at ages 6 and 12, using path analysis. The total effect of maternal education on both measures of gingival bleeding was approximately 0.13 (*P* < .05). The effect was neither mediated by toothbrushing (indirect effect about 0.1%; *P* > .05) nor by dental visit (indirect effect about 1.5%; *P* > .05). The direct effect of maternal education at child's birth on gingival bleeding represented almost 100% of the total effect (*P* < .01). In Figure 3A,B, it is possible to note that maternal education had a direct effect on gingival bleeding outcomes independently of the mediators. The proposed model presented a satisfactory fit for all observed parameters, as described in Table 3.

**Table 3:** Standardized direct, indirect and total effects of maternal schooling on gingival bleeding

Exposure‐mediator outcome	Direct effect (% of total effects)	Indirect effect (% of total effects)	Total effects
Dichotomous gingival bleeding (more than 5 teeth)^a^			
Maternal schooling‐toothbrushing‐gingival bleeding	0.13 (99.9)**	0.0001 (0.1)^ns^	0.13*
Maternal schooling‐dental visit‐gingival bleeding	0.13 (91.5)**	0.002 (1.5)^ns^	0.13*
Proportion of gingival bleeding^b^			
Maternal schooling‐toothbrushing‐gingival bleeding	0.14 (98.2)**	0.0001 (0.1)^ns^	0.14*
Maternal schooling‐dental visit‐gingival bleeding	0.14 (90.7)**	0.001 (0.7)^ns^	0.14*

ns, not significant (*P* > .05).

^a^Dichotomous gingival bleeding (more than 5 teeth): χ^2^ = 0.319; CFI = 0.99; TLI = 0.98; RMSEA = 0.02 (90% CI = 0.01;0.05).

^b^Proportion of gingival bleeding: χ^2^ = 0.294; CFI = 0.98; TLI = 1.04; RMSEA = 0.02 (90% CI = 0.01;0.05).

*P < .05; **P = .001.

### 3.1 Sensitivity analysis

Table S2 displays the scenarios when the controlled direct effect of maternal schooling on prevalence of gingival bleeding would be invalidated. To eliminate the CDE, the probability of *U* being present must be 65% (P1‐P2) higher among exposed compared with nonexposed, and the effect of *U* on the outcome to be increased at least 3 times (γ). We could not find a hypothetical scenario where the controlled direct effect of maternal schooling on the proportion of gingival bleeding would be eliminated.

## 4 DISCUSSION

There is a direct effect of maternal education on adolescents' gingival bleeding which is not mediated by oral health‐related behaviours confirmed either using MSM or path analysis. These findings suggest that an alternative explanation for the effect of socioeconomic conditions, such as maternal education, on adolescents' gingival health exists beyond oral health‐related behaviour. In addition, the findings of our study also suggest that early life exposure to detrimental socioeconomic conditions—in this case low maternal education—may have a harmful impact on adolescent's gingival health. However, it is important to mention that the reason for dental visit due to periodontal conditions, such as gingival bleeding, is uncommon in childhood and adolescence. This may explain the lack of mediation by participants' dental visiting on the association between maternal education and gingival bleeding.

Before we discuss our findings, it is appropriate to examine the study's limitations and strengths. Study's limitations include the relatively small sample size and the lack of examiner reliability measures for this outcome. Although the sample size had statistical power to identify relevant associations, the precision of our estimates was affected, as noted by wide 95% confidence intervals. Examiner reliability measurement implies in repeated gingival assessments in the same individuals by different examiners which is unrealistic given that the identification of gingival bleeding by one examiner may bias the subsequent examination. Also, the maximum‐likelihood estimation was used in path analysis. Even though this method provides robust estimates, the inclusion of categorical variables in the analytical model might have underestimated the magnitude of the coefficients. However, as our goal focused on the relationship between conditions and not on the magnitude of coefficients, the estimation method would not modify our findings. Despite the wide employment of path analysis in social sciences and economics, the estimates originated from this analytical approach may be more difficult for the interpretation of policy makers and health professionals than other well‐known effect measures such as risk ratio. The coefficient originated from path analysis is a correlation coefficient adjusted by its standard deviation. In fact, we admit the difficulty in understanding, and interpreting such measure. Finally, even though we have investigated the role of oral health behaviours as potential mediators, those measures are not particularly specific. It is important to recognize the potential social desirability bias in reporting the frequency of toothbrushing. Additionally, the frequency of toothbrushing does not reflect the quality of plaque control. Dental attendance in the last year also does not account for the reason why children visited a dentist, which could vary from preventive to pain‐related visits. Considering this, it is not possible to precisely estimate the contributions of oral health behaviours.

Our study has some methodological strengths such as a representative sample of a population‐based cohort study and high participation rates during follow‐up. In addition, we have assessed the effect of maternal schooling at birth on gingivitis at age 12 using two different analytical approaches recommended for assessing mediation in longitudinal data. Marginal structural models (MSM) circumvent possible collider bias created by conventional regression approach, when regressing the mediator conditioned on the exposure.^19^ The counterfactual approach considered in MSM suggests a causal interpretation between exposure and outcome, when certain conditions, such as positivity and exchangeability, are satisfied. Those conditions might be violated if the mean value of stabilized weight was far from 1.00. In our study, those values were 1.00; thus, it is possible to assume that MSM assumptions were properly satisfied. Marginal structural model is a useful approach for observational studies, when exposures cannot be randomly allocated in a controlled trial, such as maternal education at the time of child's birth. Path analysis is also appropriate to deal with mediation in longitudinal studies. Even though this approach does not employ the counterfactual scenario, it provides estimates that may suggest a causal interpretation. The parameters of fit observed in the final measurement model assure the robustness of our findings obtained with this analytical approach. It is worth mentioning that both approaches MSM and path analysis have revealed similar results, indicating a direct association between low maternal education at child's birth and gingival bleeding at age 12, independently of the analytical approach employed.

Three different pathways to ill‐health have been proposed, and these include material factors, psychosocial and behavioural factors. These proposed pathways may have acted in isolation or in combination^31^ which may explain our findings. Nevertheless, there is a direct association between maternal education and adolescents' gingival bleeding suggesting something beyond oral health‐related behaviour. Blane al.^32^ stated that explanations of socioeconomic inequalities in health have been biased towards behavioural explanations. This was corroborated by Watt^33^ for oral health; he stated that policy interventions aimed at changes in individuals' behaviours using a biomedical approach needed to be shifted towards more distal determinants of oral health through a combination of public health strategies. The differentiation between behavioural and alternative theories to explain socioeconomic inequalities in health is relevant for academic and policy reasons given that the understanding of the causal determinants of ill‐health should indicate the most appropriate policy to prevent or control it.

There is a body of evidence indicating that lower levels of maternal literacy are associated with worse health children's early and late outcomes.^14^, ^34^, ^35^, ^36^, ^37^ Victora et al^34^ examined the role of maternal education in several children's health conditions in a Brazilian birth cohort and found that this association was partly independent from other socioeconomic factors and that maternal care is more important than the biological characteristics of the mother. Cheng and Li^14^ investigated the influence of maternal education on the health of young children using a large sample of adopted children from China and found that the mother's education is an important determinant of the health of adopted children even after controlling for income and other socioeconomic characteristics. The effect of the mother's education on the adopted children was similar to that on the own birth sample, suggesting that the main effect of the maternal education on child health is in postnatal nurturing, beyond biology. The explanation for the link between low maternal education and poor children's health outcomes is that whose mothers have limited education tend to have lower levels of cognitive or socioemotional functioning and less academic achievement than other children.^35^

There are very few studies to compare our findings. However, our findings corroborate those of a previous study in the same topic^1^ using a more robust study design and statistical analysis, which took into account the chronological order of the life course events, along with dealing with mediation using MSM and path analysis.

Our findings have some theoretical and practical implications. Firstly, they revealed that both analytical approaches MSM and path analysis have consistent results, indicating a direct association between low maternal schooling at child's birth and gingival bleeding at age 12. Furthermore, the pattern of oral health behaviours does not explain the association between mothers' education and adolescents' gingival bleeding. Consequently, individual‐based approaches focused on oral health‐related behaviours tend to fail to prevent gingival bleeding.
