# Supplementary material for: Oral health‐related behaviours do not mediate the effect of maternal education on adolescents' gingival bleeding: A birth cohort study
Source: Community Dent Oral Epidemiol. 2017 Nov 27;46(2):169–77. doi: 10.1111/cdoe.12350 (PMC5887883; doi:10.1111/cdoe.12350)
Supplement: Supplementary file 1 [file CDOE-46-169-s001.docx]

**Table S1**

Multiplicative interaction between maternal schooling at child’s birth and dental visit and frequency of toothbrushing at age 12. 1993 Pelotas birth cohort; Pelotas; Brazil.

| **Parameter** | **Estimate** | **95%CI** | **Std. Error** | **P-value** |
| --- | --- | --- | --- | --- |
| *Dichotomous gingival bleeding (more than 5 teeth)* |  |  |  |  |
| Maternal education (X) | 1.22 | 0.57;1.86 | 0.33 | <0.001 |
| Frequency of toothbrushing (M1) | 1.58 | -0.15;3.18 | 0.82 | 0.061 |
| X#M1 | -0.79 | -2.48;0.89 | 0.86 | 0.356 |
| Intercept | -1.29 | -1.87;-0.73 | 0.29 | <0.001 |
|  |  |  |  |  |
| *Dichotomous gingival bleeding (more than 5 teeth)* |  |  |  |  |
| Maternal education (X) | 1.27 | 0.63;1.91 | 0.32 | <0.001 |
| Dental visit at age 12 (M2) | 1.02 | -0.41;2.45 | 0.73 | 0.163 |
| X#M2 | -0.53 | -2.08;1.02 | 0.79 | 0.503 |
| Intercept | -1.24 | -1.82;-0.68 | 0.29 | <0.001 |
|  |  |  |  |  |
| *Proportion of gingival bleeding* |  |  |  |  |
| Maternal education (X) | 1.10 | 0.41;1.80 | 0.35 | 0.002 |
| Frequency of toothbrushing (M1) | 1.80 | -0.09;3.70 | 0.96 | 0.062 |
| X#M1 | -1.09 | -3.08;0.88 | 1.01 | 0.277 |
| Intercept | -1.60 | -2.25;-0.94 | 0.33 | <0.001 |
|  |  |  |  |  |
| *Proportion of gingival bleeding* |  |  |  |  |
| Maternal education (X) | 1.07 | 0.37;1.77 | 0.36 | 0.003 |
| Dental visit at age 12 (M2) | 0.86 | -0.77;2.48 | 0.83 | 0.301 |
| X#M2 | -0.19 | -1.94;1.56 | 0.89 | 0.835 |
| Intercept | -1.51 | -2.15;-0.87 | 0.33 | <0.001 |

Additive Interaction between maternal schooling at child’s birth and dental visit and frequency of toothbrushing ate age 12. 1993 Pelotas birth cohort; Pelotas; Brazil.

| **Parameter** | **Estimate** | **95%CI** | **Std. Error** | **P-value** |
| --- | --- | --- | --- | --- |
| *Dichotomous gingival bleeding (more than 5 teeth)* |  |  |  |  |
| RERI  Maternal education# Frequency of toothbrushing | -1.97 | -13.52;9.58 | 5.89 | 0.738 |
|  |  |  |  |  |
| *Dichotomous gingival bleeding (more than 5 teeth)* |  |  |  |  |
| RERI  Maternal education# Dental visit at age 12 | 1.45 | -3.48;6.38 | 2.51 | 0.565 |
|  |  |  |  |  |
| *Proportion of gingival bleeding* |  |  |  |  |
| RERI  Maternal education# Frequency of toothbrushing | -1.25 | -3.80;1.30 | 1.30 | 0.337 |
|  |  |  |  |  |
| *Proportion of gingival bleeding* |  |  |  |  |
| RERI  Maternal education# Dental visit at age 12 | 0.02 | -1.33;1.38 | 0.69 | 0.974 |

*Interaction analysis*

In mediation analysis, one should consider that the outcome of interest is influenced by the exposure and by the mediator; also that the mediator is also influenced by the exposure. In this scenario, the outcome may be related to the combination of two conditions: the exposure and the mediator. Thus, the joint effect of exposure and mediator on the outcome should be investigated. Interaction can be assessed on different scales: additive or multiplicative. The fact that interaction can be assessed on different scales and that interaction is scale-dependent raises the question on which scale interaction should be assessed. The literature has pointed that it is recommended to present both additive and multiplicative measures of interaction. However, only measures of multiplicative interaction are often reported. Since multiplicative interaction is directly obtained, its reporting is done because of convenience, rather than because thought has been given to which measure should be reported^1^.

Additive interaction is of relevance for assessing public health relevance of an intervention, since it is useful for targeting subpopulations for which the intervention is most effective. In addition, tests for detecting additive interaction are usually more powerful than tests for multiplicative interaction; thus, it is more easily detectable. The multiplicative scale is also of importance. Firstly, it is easier to fi multiplicative models; and most standard software can provide the most natural scale on which to assess interpretation for such models. Some authors have proposed that the multiplicative scale is better suited to “assess causality” due to the relative effect measure provided.

Since both scales present specificities regarding their application, many authors suggest reporting both additive and multiplicative interaction^1, 2^.

**References**

1. Knol MJ, VanderWeele TJ. Recommendations for presenting analyses of effect modification and interaction. *Int J Epidemiol* 2012;41:514-520.

2. Botto LD, Khoury MJ. Commentary: Facing the challenge of gene-environment interaction: The two-by-four table and beyond. *Am J Epidemiol* 2001;153:1016-1020.
