# Supplementary material for: Oral health‐related behaviours do not mediate the effect of maternal education on adolescents' gingival bleeding: A birth cohort study
Source: Community Dent Oral Epidemiol. 2017 Nov 27;46(2):169–77. doi: 10.1111/cdoe.12350 (PMC5887883; doi:10.1111/cdoe.12350)
Supplement: Supplementary file 2 [file CDOE-46-169-s002.docx]

**Table S2**

Sensitivity analysis for unmeasured confounder considering the estimates of exposure on outcomes.

| $\boldsymbol{\gamma}$ | **P1** | **P2** | **P1-P2** | **RR** | $\boldsymbol{\gamma}$ | **P1** | **P2** | **P1-P2** | **RR** |
| --- | --- | --- | --- | --- | --- | --- | --- | --- | --- |
| **3** | 0.7 | 0.05 | 0.65 | 2.18 | **4** | 0.4 | 0.02 | 0.38 | 2.07 |
| **3** | 0.8 | 0.05 | 0.75 | 2.36 | **4** | 0.5 | 0.02 | 0.48 | 2.35 |
| **3** | 0.9 | 0.05 | 0.85 | 2.54 | **4** | 0.6 | 0.02 | 0.58 | 2.64 |
| **3** | 0.8 | 0.1 | 0.7 | 2.17 | **4** | 0.7 | 0.02 | 0.68 | 2.92 |
| **3** | 0.9 | 0.1 | 0.8 | 2.33 | **4** | 0.8 | 0.02 | 0.78 | 3.20 |
|  |  |  |  |  | **4** | 0.9 | 0.02 | 0.88 | 3.50 |
|  |  |  |  |  | **4** | 0.5 | 0.05 | 0.45 | 2.17 |
|  |  |  |  |  | **4** | 0.6 | 0.05 | 0.55 | 2.44 |
|  |  |  |  |  | **4** | 0.7 | 0.05 | 0.65 | 2.70 |
|  |  |  |  |  | **4** | 0.8 | 0.05 | 0.75 | 2.95 |
|  |  |  |  |  | **4** | 0.9 | 0.05 | 0.85 | 3.22 |
|  |  |  |  |  | **4** | 0.6 | 0.1 | 0.5 | 2.15 |
|  |  |  |  |  | **4** | 0.7 | 0.1 | 0.6 | 2.38 |
|  |  |  |  |  | **4** | 0.8 | 0.1 | 0.7 | 2.61 |
|  |  |  |  |  | **4** | 0.9 | 0.1 | 0.8 | 2.84 |
|  |  |  |  |  | **4** | 0.8 | 0.2 | 0.6 | 2.12 |
|  |  |  |  |  | **4** | 0.9 | 0.2 | 0.7 | 2.31 |

**γ:** conditional increase in the risk of unfavorable periodontal outcomes;

**P1:** Prevalence in exposed; **P2:** Prevalence in non-exposed; **P1-P2:** Calculated differences in the probability of U; **RR:** Estimated effect.

In all hypothesized scenarios, the presence of unmeasured confounder not included in the analytical models would nullify only the effect of maternal schooling at birth on the proportion of gingival bleeding at age 12. No hypothesized scenario would eliminate the effect of low maternal schooling at birth on the presence of gingival bleeding (more than 5 teeth) at age 12.
