# Supplementary material for: Oral health‐related behaviours do not mediate the effect of maternal education on adolescents' gingival bleeding: A birth cohort study
Source: Community Dent Oral Epidemiol. 2017 Nov 27;46(2):169–77. doi: 10.1111/cdoe.12350 (PMC5887883; doi:10.1111/cdoe.12350)
Supplement: Supplementary file 3 [file CDOE-46-169-s003.docx]

***Appendix S1***

***Steps used for estimating stabilized inverse probability weights for Marginal Structural Model***

Baseline confounders were accounted for estimating the stabilized inverse probability weight (S*W=*$W_{i}^{X}*W_{i}^{M1}* W_{i}^{M2}$) where:

The stabilized inverse probability weight of the exposure was estimated based on the following formula:

$$W_{i}^{X}=\frac{P(X=x_{i})}{P(X=x_{i}|C=c_{i})}$$

where *x_i_,* *m_i_* and *c_i_* are the actual values of exposure, mediator and covariates for individual *i.*

Stabilized weights of mediators at 6 years-old were estimated as follows:

$$W_{i}^{M}(6yo)=\frac{P(M(6yo)=m_{i}(6yo)|X=x_{i})}{P(M(6yo)=m_{i}(6yo)|X=x_{i},L'=l_{i}\left( birth \right), L''= l_{i}(4yo), C=c_{i})}$$

Stabilized weights of the mediators at 12-years-old were estimated as follows:

$$W_{i}^{M}(12yo)=\frac{P(M(12yo)=m_{i}(12yo)|X=x_{i}, M=m_{i}(6yo))}{P(M(12yo)=m_{i}(12yo)|X=x_{i}, M=m_{i}\left( 6yo \right), L'=l_{i}\left( birth \right), L''= l_{i}\left( 4yo \right), L'''=l_{i}\left( 11yo \right),C=c_{i})}$$

where *x_i_(t),* *m_i_(t)* are the actual values of the exposure and the mediator; and *c_i_* represents the baseline covariates for individual *i*. For the mediator weight at time *t*, we have the mediator value that was in fact present at time t conditional on the individual’s past

mediator history, time-varying covariate history, and baseline covariates.

Final stabilized weight was obtained taking a product of these weights across the time:

$${SW}_{i}= W_{i}^{X}* W_{i}^{M1}\left( 6yo \right)* W_{i}^{M1}\left( 12yo \right)* W_{i}^{M2}\left( 6yo \right)* W_{i}^{M2}\left( 12yo \right)$$

The distribution of final stabilized weights was: mean= 1.00; range= 0.34-5.38; Interquartile range (IQR)= 0.61-1.21. All analyses were conducted in the software Stata 14.0 (StataCorp.; College Station, TX, USA).
