# Supplementary material for: Oral health‐related behaviours do not mediate the effect of maternal education on adolescents' gingival bleeding: A birth cohort study
Source: Community Dent Oral Epidemiol. 2017 Nov 27;46(2):169–77. doi: 10.1111/cdoe.12350 (PMC5887883; doi:10.1111/cdoe.12350)
Supplement: Supplementary file 4 [file CDOE-46-169-s004.docx]

**Appendix S2**

We used the following model given by VanderWeele to calculate the bias introduced by U that could invalidate the controlled direct effect:

$$Bias {CDE}_{X, X^{*}|c}^{RR}\left( m \right)=\frac{1+\left( \gamma-1 \right)P1(U=1|X, M,C)}{1+\left( \gamma-1 \right)P2(U=1|X^{*},M,C)}$$

For conducting this analysis, we need to assume two aspects: 1) the prevalence of U; and 2) the effect of U on the outcome. We also assumed there is no relative excess risk due to interaction between exposure and U. The parameters of U, such as γ (conditional increase in risk of gingival bleeding), P1 (P(U=1|X, M, C)), and P2 (P(U=1|X*, M, C)) were specified according to the literature.

**Reference:**

VanderWeele TJ. Bias formulas for sensitivity analysis for direct and indirect effects. Epidemiology. 2010;21(4):540-551.
